# Intravenous Immunoglobulin for Refractory Streptococcal Toxic Shock Syndrome With Purpura Fulminans: A Case Report and Literature Review

**DOI:** 10.7759/cureus.104462

**Published:** 2026-02-28

**Authors:** Rakshit Shetty, Shyam Kiran Gandam Venkata, Sai Sruthi Bhuram, Sudeep Chakravarthy Bhuram, Mahi Chanpura

**Affiliations:** 1 Department of Critical Care Medicine, Springfield Clinic, Springfield, USA; 2 Department of Pulmonary and Critical Care Medicine, Southern Illinois University School of Medicine, Springfield, USA

**Keywords:** critical illness, immunomodulatory therapy, intravenous immunoglobulin, invasive group a streptococcal infection, multiorgan failure, purpura fulminans, refractory septic shock, streptococcal toxic shock syndrome, streptococcus pyogenes, toxin-mediated shock

## Abstract

Streptococcal toxic shock syndrome (STSS) is a rare but life-threatening condition characterized by rapid progression to septic shock and multiorgan dysfunction driven by toxin-mediated immune activation. We report the case of a previously healthy 39-year-old man who presented with acute encephalopathy and rapidly deteriorated into refractory septic shock requiring mechanical ventilation, maximal vasopressor support, and continuous renal replacement therapy. Blood cultures identified *Streptococcus pyogenes*, and the clinical course was complicated by severe metabolic acidosis, acute liver injury, thrombocytopenia, and the development of purpura fulminans, consistent with STSS. Despite early broad-spectrum antimicrobial therapy transitioned to antitoxin-directed antibiotics and aggressive supportive management, the patient demonstrated persistent hemodynamic instability and progressive multiorgan failure. Adjunctive intravenous immunoglobulin (IVIG) was administered as part of a multidisciplinary treatment strategy in the setting of refractory shock and suspected ongoing toxin-mediated injury. Following IVIG therapy, the patient exhibited gradual hemodynamic improvement with decreasing vasopressor requirements and stabilization of organ function. This case illustrates the diagnostic and therapeutic uncertainty in toxin-mediated septic shock and demonstrates how IVIG was considered as a rescue adjunct in the setting of persistent hemodynamic instability despite optimal standard therapy. The report aims to contextualize clinical decision-making rather than attribute causality to a single intervention.

## Introduction

Streptococcal toxic shock syndrome (STSS) is a fulminant, toxin-mediated illness caused by invasive infection with *Streptococcus pyogenes* and is characterized by the rapid onset of shock, multiorgan dysfunction, and high mortality despite modern critical care support. The pathogenesis of STSS is driven by streptococcal superantigens that induce widespread, non-antigen-specific T-cell activation, resulting in massive cytokine release, endothelial injury, and circulatory collapse [1]. Even with prompt recognition and aggressive management, reported mortality rates remain substantial, particularly in patients presenting with refractory shock or purpura fulminans.

Current management strategies for STSS emphasize early hemodynamic resuscitation, prompt source control, and antimicrobial therapy that includes a beta-lactam agent combined with clindamycin to suppress toxin production and mitigate the superantigen-mediated inflammatory response [2]. However, antimicrobial therapy alone does not neutralize circulating exotoxins already present at the time of clinical deterioration [3,4].

Adjunctive intravenous immunoglobulin (IVIG) has been proposed as a therapeutic option in severe STSS based on its ability to neutralize streptococcal superantigens and exotoxins and modulate the host immune response. Although the mechanistic rationale is strong, clinical evidence supporting IVIG use remains limited, consisting primarily of a small randomized trial and observational studies with inherent heterogeneity [1,3]. As a result, major clinical guidelines acknowledge the potential role of IVIG in selected severe cases while stopping short of recommending its routine use. This case report describes a patient with refractory STSS complicated by purpura fulminans and multiorgan failure, highlighting the clinical course, management challenges, and considerations surrounding adjunctive immunomodulatory therapy.

Importantly, current literature does not provide definitive evidence of efficacy. Available data consist largely of small randomized trials and observational studies with confounding by severity, and therefore, IVIG should be interpreted as a rescue adjunct rather than an established therapy.

## Case presentation

A 39-year-old previously healthy man presented with acute-onset altered mental status, progressive lethargy, and recurrent vomiting. He was unable to provide his history on arrival. According to the family, symptoms began abruptly one day prior without associated diarrhea. He had been evaluated at an urgent care facility where testing for COVID-19, influenza, and streptococcal infection was reportedly negative. A close household contact had recently been treated for streptococcal pharyngitis. There was no recent travel or trauma. No focal necrotizing soft tissue source was identified on physical examination. Imaging did not reveal a drainable focus, and surgical consultation did not identify an indication for operative exploration.

On presentation, the patient appeared acutely ill and lethargic but arousable. Vital signs were notable for sinus tachycardia with heart rates in the 140s and a widened pulse pressure with blood pressure of 120/40 mmHg. Neurologic examination revealed no focal deficits. Cardiopulmonary and abdominal examinations were initially unremarkable. Within hours of admission, the patient experienced rapid neurologic deterioration requiring endotracheal intubation for airway protection. He developed progressive hypotension refractory to fluid resuscitation and required escalating doses of multiple vasopressor agents.

The patient was diagnosed with septic shock complicated by acute encephalopathy, acute hypoxic respiratory failure, acute kidney injury, severe metabolic acidosis, coagulopathy, and hypoglycemia. Lumbar puncture was attempted but was unsuccessful due to body habitus. Continuous renal replacement therapy was initiated for renal failure and metabolic derangements. Persistent hypoglycemia required continuous dextrose infusion. Key laboratory abnormalities at peak illness severity are summarized in Table 1. Blood cultures subsequently grew *S. pyogenes*, confirming invasive group A streptococcal bacteremia. These abnormalities met criteria for severe streptococcal toxic shock syndrome and reflected profound tissue hypoperfusion and organ dysfunction.

**Table 1:** Laboratory abnormalities during acute illness Laboratory values represent the most abnormal measurements recorded during hospital days 1–3 (acute shock phase), not solely admission values.

Laboratory Parameter	Peak or Worst Value	Reference Range
White blood cell count	45 ×10³/µL	4.0–11.0 ×10³/µL
Platelet count	73 ×10³/µL	150–400 ×10³/µL
Creatinine	3.7 mg/dL	0.7–1.3 mg/dL
Aspartate aminotransferase	3,445 U/L	10–40 U/L
Alanine aminotransferase	1,616 U/L	7–56 U/L
International normalized ratio	2.5	0.9–1.1
Lactate	11 mmol/L	0.5–2.2 mmol/L

Broad-spectrum antimicrobial therapy was initiated and subsequently narrowed to beta-lactam therapy in combination with clindamycin. Despite aggressive resuscitation, stress-dose corticosteroids, continuous renal replacement therapy, and escalating vasopressor support, the patient developed progressive multiorgan dysfunction. On hospital day 2, he manifested diffuse purpura fulminans with blistering and discoloration of the digits and lower extremities, consistent with toxin-mediated microvascular thrombosis.

On hospital day 3, given persistent vasopressor dependence and concern for ongoing toxin-mediated disease despite appropriate antimicrobial therapy and supportive care, adjunctive intravenous immunoglobulin was administered at a cumulative dose of approximately 1.1 g/kg.

During hospital days 3 through 5, vasopressor requirements initially increased before subsequently trending downward following intravenous immunoglobulin administration, coinciding with hemodynamic stabilization. Renal function remained supported with continuous renal replacement therapy, and no intrinsic renal recovery was inferred during this period. The patient ultimately survived but sustained ischemic complications requiring partial limb amputations. Renal function improved following discontinuation of continuous renal replacement therapy. The overall clinical course and major interventions are summarized in Table 2. The timeline emphasizes the persistence of shock despite appropriate antimicrobial therapy prior to consideration of adjunctive therapy.

**Table 2:** Clinical course and major interventions Trends in vasopressor requirements reflect hemodynamic support needs and do not imply causality.

Hospital Day	Clinical Course
Days 1–2	Rapid neurologic decline requiring intubation; refractory septic shock with escalating vasopressor requirements; initiation of continuous renal replacement therapy
Days 3–5	Initial increase in vasopressor requirements followed by subsequent downward trend after intravenous immunoglobulin administration
After day 5	Discontinuation of vasopressors; continued continuous renal replacement therapy; stabilization of metabolic parameters
Outcome	Survival with ischemic limb complications requiring partial amputations; renal recovery after discontinuation of continuous renal replacement therapy

## Discussion

GAS toxic shock syndrome (TSS) is a fulminant, life-threatening illness caused by *S. pyogenes*, characterized by rapid progression to shock and multiorgan failure. The pathogenesis is driven by streptococcal superantigens that induce widespread T-cell activation and a profound cytokine surge, resulting in distributive shock, endothelial injury, and tissue hypoperfusion. GAS TSS often occurs in previously healthy individuals and is frequently associated with invasive soft tissue infections, including necrotizing fasciitis. Early recognition is critical, as mortality remains high despite appropriate therapy. The diagnosis is primarily clinical and is based on established criteria encompassing hypotension and multisystem involvement, as outlined in Table 3. In this case, the patient fulfilled confirmed STSS criteria based on hypotension, multiorgan involvement, and isolation of group A Streptococcus from blood cultures.

**Table 3:** Clinical diagnostic criteria for streptococcal toxic shock syndrome (STSS) Adapted from the Centers for Disease Control and Prevention (CDC) Streptococcal Toxic Shock Syndrome (STSS) 2010 Case Definition [5] and the Infectious Diseases Society of America (IDSA) Skin and Soft Tissue Infection Guidelines (2014) [2] STSS, streptococcal toxic shock syndrome; ULN, upper limit of normal; ARDS, acute respiratory distress syndrome.

Diagnostic Domain	Criterion
Hemodynamic instability	Systolic blood pressure ≤90 mmHg in adults, or <5th percentile for age in children <16 years
Multiorgan involvement *(≥2 required)*	
Renal involvement	Serum creatinine ≥2.0 mg/dL (177 μmol/L) in adults; ≥2 times upper limit of normal (ULN) for age in children; or ≥2 times baseline in patients with chronic kidney disease
Coagulation abnormality	Platelet count ≤100,000/mm³ (≤100 × 10⁶/L) or disseminated intravascular coagulation (prolonged coagulation times, hypofibrinogenemia, and/or fibrin degradation products)
Hepatic involvement	Alanine aminotransferase, aspartate aminotransferase, or total bilirubin ≥2 times ULN for age; or ≥2 times baseline in patients with preexisting liver disease
Pulmonary involvement	Acute respiratory distress syndrome
Cutaneous findings	Diffuse erythematous macular rash, with or without subsequent desquamation
Soft tissue involvement	Evidence of necrotizing soft tissue infection (eg, necrotizing fasciitis, myositis, or gangrene)
Microbiologic classification	
Probable STSS	Meets clinical criteria without an alternative explanation, with isolation of group A Streptococcus from a nonsterile site (eg, throat, skin lesion, vagina)
Confirmed STSS	Meets clinical criteria with isolation of group A Streptococcus from a normally sterile site (eg, blood, cerebrospinal fluid, pleural fluid, peritoneal fluid, joint fluid, tissue biopsy, or surgical specimen)
Recommended cultures	At least two sets of blood cultures plus cultures from clinically relevant suspected sites of infection

The differential diagnosis of GAS TSS includes other infectious and inflammatory conditions that present with fever, shock, rash, and multiorgan dysfunction. Distinguishing features such as exposure history, laboratory patterns, and microbiologic data are essential to differentiate GAS TSS from these mimics. A structured comparison of key alternative diagnoses and their distinguishing characteristics is summarized in Table 4.

**Table 4:** Differential diagnosis of invasive group A streptococcal toxic shock syndrome (TSS) This table summarizes key infectious and non-infectious conditions that may present with fever, shock, rash, and multiorgan dysfunction and can clinically mimic streptococcal toxic shock syndrome. Distinguishing features and diagnostic approaches are derived from established clinical descriptions and guidelines, including Walker et al., 2014 [1], Centers for Disease Control and Prevention (CDC) Streptococcal Toxic Shock Syndrome (STSS) 2010 [5], Infectious Diseases Society of America (IDSA) Skin and Soft Tissue Infection Guidelines (2014) [2], and Carapetis et al., 2005 [6]. TSS, toxic shock syndrome; GAS, group A Streptococcus; WBC, white blood cell count; CSF, cerebrospinal fluid; MIS, multisystem inflammatory syndrome; CNS, central nervous system.

Condition	Key clinical Features	Distinguishing Features vs TSS	Diagnostic Approach
Staphylococcal TSS	Rapid onset fever, hypotension, diffuse erythematous rash, multiorgan dysfunction	Often occurs in otherwise healthy individuals; associated with tampon use, recent surgery, or skin/soft-tissue infection	Clinical and laboratory criteria
Gram-negative sepsis	Fever, hypotension, respiratory failure	Renal failure typically follows hypotension (contrast: renal dysfunction often precedes hypotension in GAS TSS)	Blood cultures
Typhoid fever	Prolonged fever, abdominal pain, relative bradycardia	Normal or low WBC count; pulse–temperature dissociation	Blood, stool, or bone-marrow cultures
Rocky Mountain spotted fever (RMSF)	Fever, headache, rash	Prominent headache; petechial rash (vs diffuse erythema in TSS); rash more common	Clinical diagnosis ± serology
Meningococcemia	Sudden fever, nausea, vomiting, petechial rash	Frequent meningitic symptoms; rash typically petechial	Blood and CSF cultures
Streptococcus pneumoniae infection	Fever, respiratory symptoms, lobar pneumonia, empyema	Primary pulmonary focus common; bacteremia may occur without pneumonia (especially in children)	Blood or respiratory cultures
Kawasaki disease	Prolonged fever, mucocutaneous inflammation, lymphadenopathy	Occurs primarily in children; vasculitic features	Clinical criteria supported by labs
COVID-19–associated MIS	Fever, GI symptoms, myocarditis, shock	Elevated inflammatory markers; history of SARS-CoV-2 exposure or infection	Clinical evaluation, SARS-CoV-2 testing
Leptospirosis	Fever, rigors, myalgia, headache, abdominal pain	Conjunctival suffusion is characteristic and uncommon in TSS	Serologic testing
Heat stroke	Core temperature >40°C, CNS dysfunction	Clear history of heat exposure; hyperthermia is primary abnormality	Clinical diagnosis

Mechanism and pathophysiology of TSS

TSS is a fulminant toxin-mediated illness most commonly caused by *S. pyogenes* or *Staphylococcus aureus*. The hallmark of the disease is the production of bacterial superantigens, such as streptococcal pyrogenic exotoxins, that bypass conventional antigen processing and directly activate large populations of T lymphocytes by cross-linking major histocompatibility complex class II molecules with T-cell receptors [1,4]. This interaction leads to massive polyclonal T-cell activation and an uncontrolled cytokine surge, including tumor necrosis factor-α, interleukin (IL)-1β, IL-6, and interferon-γ, resulting in distributive shock, endothelial injury, capillary leak, and multiorgan dysfunction [7].

**Figure 1:**
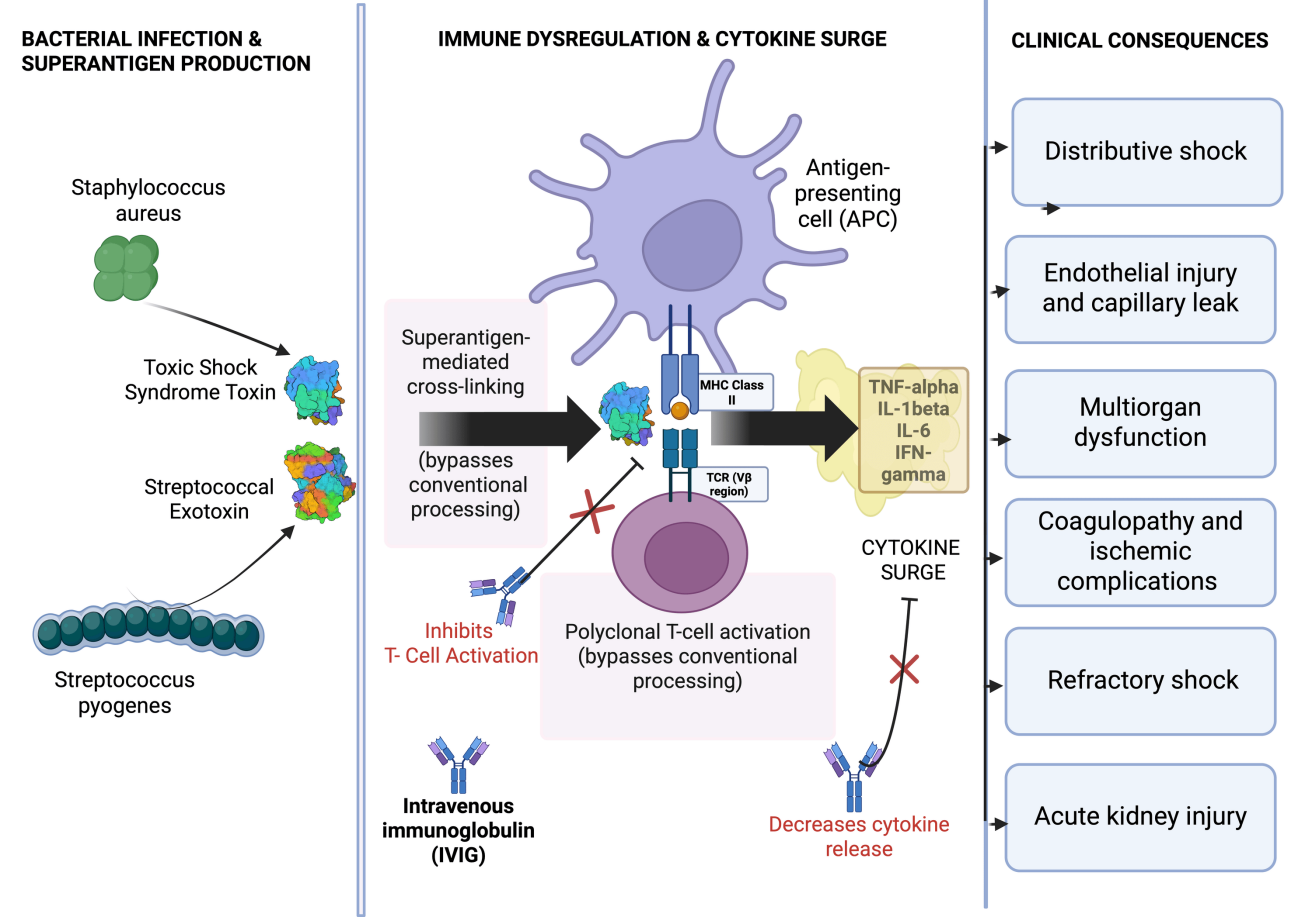
Pathophysiology of superantigen-mediated TSS and mechanisms of IVIG modulation *Streptococcus pyogenes* and *Staphylococcus aureus* produce superantigenic exotoxins that bypass conventional antigen processing by directly cross-linking MHC class II molecules on APCs with the variable β (Vβ) region of the TCR. This interaction results in widespread, non-specific T-cell activation and a subsequent cytokine surge characterized by elevated levels of TNF-α, IL-1β, IL-6, and IFN-γ. The downstream clinical consequences include distributive shock, endothelial injury with capillary leak, multiorgan dysfunction, coagulopathy with ischemic complications, refractory shock, and acute kidney injury. IVIG is depicted at multiple points in the pathway, highlighting its proposed mechanisms of action, including neutralization of circulating superantigens, inhibition of T-cell activation, and attenuation of proinflammatory cytokine release. TSS, toxic shock syndrome; APC, antigen-presenting cell; IVIG, intravenous immunoglobulin; IL, interleukin; TCR, T-cell receptor; TNF-α, tumor necrosis factor-α; IFN-γ, interferon-γ; MHC, major histocompatibility complex Image Credit: Authors; created using BioRender.com (licensed for publication).

In STSS, this immune dysregulation often coincides with invasive infection, accelerating progression to refractory shock, acute kidney injury, coagulopathy, and ischemic complications such as purpura fulminans. Purpura fulminans represents a severe manifestation of sepsis-associated coagulopathy, driven by endothelial injury, depletion of protein C and other natural anticoagulant pathways, and diffuse microvascular thrombosis, resulting in rapidly progressive tissue ischemia and necrosis. Mortality remains high despite advances in critical care, emphasizing the importance of early recognition and aggressive intervention [6]. Figure 1 depicts the pathophysiologic mechanisms of toxic shock syndrome, highlighting superantigen-mediated immune activation, cytokine surge, and downstream clinical consequences. IVIG theoretically acts upstream at the level of superantigen-mediated immune activation rather than directly reversing established shock physiology.

Standard-of-care management

Current guidelines from the Infectious Diseases Society of America (IDSA) and the Centers for Disease Control and Prevention (CDC) emphasize that early standard-of-care management remains the most evidence-supported intervention in STSS [2,7]. Key elements include prompt hemodynamic resuscitation, early surgical source control when indicated, and targeted antimicrobial therapy.

Antibiotic therapy for STSS consists of a beta-lactam agent active against *S. pyogenes* combined with clindamycin. Clindamycin is recommended because it suppresses bacterial protein synthesis and exotoxin production independent of bacterial growth phase [8]. Observational cohort studies have demonstrated improved survival and reduced disease severity in invasive GAS infections when clindamycin is used as adjunctive therapy [9]. Additional clinical data further support the association between clindamycin use and improved outcomes in STSS [10]. Supportive care follows sepsis management principles, including vasopressor support, renal replacement therapy, and mechanical ventilation as needed.

Importantly, current guidelines do not endorse immunomodulatory therapies as replacements for these core interventions, and adjunctive treatments are discussed only in the context of severe, refractory disease [2].

Adjunctive therapy in STSS

This section focuses on clinical evidence and, therefore, will not re-review the previously described superantigen biology.

Because multiple interventions occurred concurrently, including antimicrobial optimization, corticosteroids, renal replacement therapy, and ongoing hemodynamic resuscitation, the observed improvement cannot be attributed to IVIG alone. Delayed clinical response to antitoxin antibiotics, such as clindamycin, or cumulative supportive care effects remain plausible alternative explanations. Therefore, the temporal association between IVIG administration and hemodynamic stabilization should be interpreted cautiously.

The biologic rationale for IVIG in STSS is based on its ability to neutralize circulating superantigens, inhibit T-cell activation, and attenuate cytokine release. Polyspecific IVIG preparations contain antibodies capable of binding streptococcal exotoxins and modulating immune responses in vitro and ex vivo, providing a plausible theoretical benefit in toxin-mediated shock [11,12].

Clinical evidence supporting IVIG use in STSS remains limited. IVIG dosing regimens reported in the literature typically range from 1 to 2 g/kg administered as a total cumulative dose. In the present case, IVIG was administered at a cumulative dose of approximately 1.1 g/kg, consistent with dosing reported in prior observational studies and clinical trials [13-15]. A small randomized controlled trial evaluating high-dose IVIG in STSS was terminated early due to slow enrollment and was underpowered to demonstrate a statistically significant mortality benefit, although mortality was numerically lower in the IVIG group [13]. Subsequent observational cohort studies reported lower mortality among patients receiving IVIG in addition to clindamycin and beta-lactam therapy; however, these findings are subject to confounding by indication and disease severity [14].

A systematic review and meta-analysis pooling randomized trials with observational studies in clindamycin-treated STSS patients demonstrated an association between IVIG administration and reduced mortality [15]. Despite this signal, the authors emphasized substantial heterogeneity, small sample sizes, and residual confounding, limiting causal inference.

Consistent with these limitations, IDSA guidelines acknowledge biologic plausibility but conclude that evidence is insufficient to recommend IVIG as standard therapy, stating that IVIG may be considered on a case-by-case basis in patients with severe STSS and refractory shock [2]. Several key uncertainties persist, including optimal timing of administration relative to shock onset and source control, appropriate dosing strategies, identification of patient subgroups most likely to benefit, and the impact of batch-to-batch variability in antitoxin activity [15].

IVIG therapy is associated with potential adverse effects, including infusion reactions, hemolysis, thromboembolic events, acute kidney injury, and volume overload. Cost and availability further limit routine use, reinforcing the need for individualized decision-making and cautious interpretation of outcomes [16].

Therapeutic plasma exchange (TPE) has been proposed as a salvage therapy in toxin-mediated shock based on its capacity to remove circulating toxins and inflammatory mediators. However, evidence supporting its use in STSS is limited to case reports and small case series, which are inherently subject to publication bias. Data from broader sepsis populations have not demonstrated consistent benefit in mortality or organ failure, and some analyses suggest potential harm or prolonged ICU stays [17]. Consequently, neither adult nor pediatric Surviving Sepsis Campaign guidelines recommend TPE as routine therapy in septic shock without thrombocytopenia-associated multiorgan failure [16].

Summary of evidence and guideline perspective

The available evidence and guideline perspectives on adjunctive immunomodulatory therapies in STSS are summarized in Table 5. The table synthesizes data from randomized trials, observational studies, meta-analyses, and expert guidelines evaluating intravenous immunoglobulin and therapeutic plasma exchange. Overall, the evidence remains limited by heterogeneity and confounding, and the table contextualizes these therapies relative to standard-of-care management.

**Table 5:** Evidence and guideline perspective on adjunctive therapies in STSS IVIG, intravenous immunoglobulin; STSS, streptococcal toxic shock syndrome; SSTI, skin and soft tissue infection; IDSA, Infectious Diseases Society of America; CDC, Centers for Disease Control and Prevention; SCCM, Society of Critical Care Medicine; SSC, Surviving Sepsis Campaign; TPE, therapeutic plasma exchange “Improved outcomes” and “reduced mortality” refer to associations observed within individual studies and do not imply causality. Guideline interpretations reflect the consensus position of the respective organizations at the time of publication and emphasize the limited quality, heterogeneity, and potential confounding of available evidence. Terms such as “suggestive association,” “hypothesis-generating,” and “inconclusive” denote evidence derived from observational studies, underpowered randomized trials, or meta-analyses with significant methodological limitations.

Evidence Source	Study Type	Key Findings	Guideline Interpretation
Darenberg et al. [13], 2003	Randomized controlled trial	Lower mortality with IVIG, not statistically significant	Underpowered; inconclusive
Kaul et al. [9], 1999	Observational cohort	Reduced mortality with IVIG + clindamycin	Suggestive association
Linnér et al. [14], 2014	Observational study	Improved outcomes with IVIG	Confounding likely
Parks et al. [15], 2018	Meta-analysis	Mortality association in clindamycin-treated STSS	Hypothesis-generating
IDSA SSTI Guidelines, 2014 [2]	Expert guideline	Biologic plausibility; insufficient evidence	IVIG may be considered selectively
CDC STSS Guidance [5]	Expert guidance	Emphasizes antibiotics and source control	IVIG not routine
SCCM/SSC Guidelines [16]	Expert guideline	No endorsement of IVIG or TPE in septic shock	Not standard of care

IVIG is not standard-of-care for STSS but may be considered as adjunctive therapy in select patients with severe, refractory disease after optimal standard management. Therapeutic plasma exchange remains investigational and should be reserved for exceptional circumstances. This case should not be interpreted as proof of efficacy. Rather, it demonstrates a clinical scenario in which IVIG was considered after failure of evidence-based therapy, reflecting real-world decision-making under uncertainty.

## Conclusions

STSS remains a rapidly progressive and frequently catastrophic illness. Early resuscitation, source control, and antitoxin antibiotics remain the cornerstone of management. Adjunctive IVIG may be considered in carefully selected patients with refractory shock; however, current evidence is insufficient to determine causality or routine benefit. This case emphasizes clinical judgment in extreme presentations rather than the therapeutic efficacy of a single intervention. Further prospective, adequately powered studies are needed to better define the role of immunomodulatory therapies in STSS.
